# The structure of the aluminium-abundant γ-brass-type Al_8.6_Mn_4.4_

**DOI:** 10.1107/S2414314621009883

**Published:** 2021-09-24

**Authors:** Qifa Hu, Bin Wen, Changzeng Fan

**Affiliations:** a State Key Laboratory of Metastable Materials Science and Technology, Yanshan University, Qinhuangdao 066004, People’s Republic of China; Benemérita Universidad Autónoma de Puebla, México

**Keywords:** crystal structure, Al–Mn, inter­metallic, γ-brass phase

## Abstract

An aluminium-abundant Al_8_Mn_5_/γ-brass-type inter­metallic with formula Al_8.6_Mn_4.4_, which is isotypic with γ-Al_8_Cr_5_ and γ-Al_8_V_5_, was discovered by high-temperature sinter­ing of an Al/Mn mixture with initial composition Al_2_Mn. Structure analysis revealed that one special position (Wyckoff site 18*h* in space group *R*  *m*) is shared by Al and Mn, with refined site occupancy factors of 0.7 and 0.3, respectively. The present low-temperature Al_8_Mn_5_-type phase crystallizes in the centrosymmetric space group *R*  *m* (No. 166), rather than *R*3*m* (No. 160) as previously reported for the same inter­metallic characterized by TEM measurements [Zeng *et al.* (2018 ▸). *Acta Mater.* **153**, 364–376].

## Abstract 2

An aluminium-abundant γ-brass phase, Al_8.6_Mn_4.4_, was synthesized by high-temperature sinter­ing, and its single-crystal structure has been determined.

## Graphical abstract

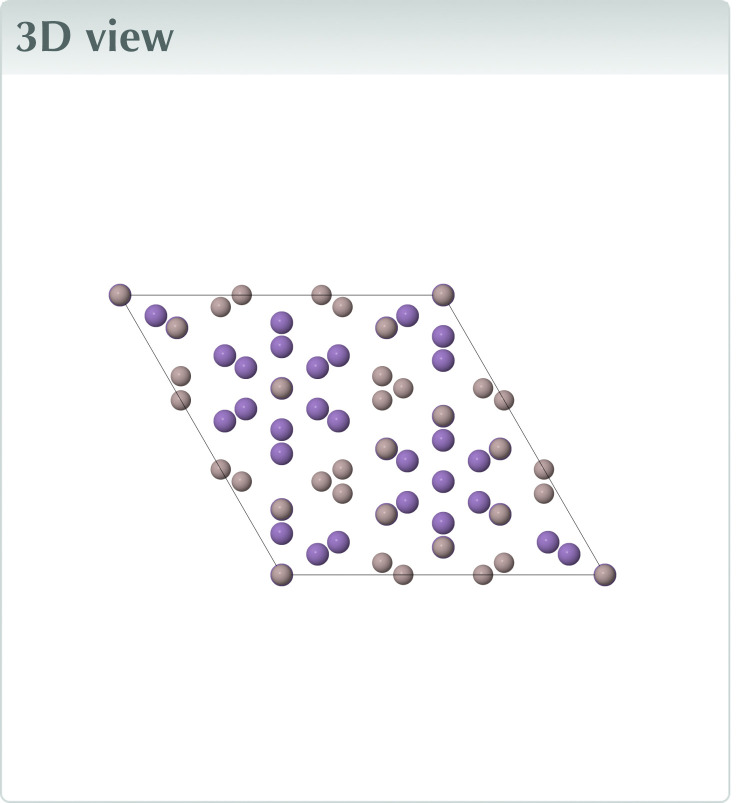

## Structure description

The γ-brasses are common phases with rhombohedral symmetry, and have been observed in many systems, including the Al–Cr, Al–Mn, Al–Cu, Ga–Cr, Ga–Mn and Ga–Fe mixtures (Bradley & Lu, 1937 ▸; Meissner *et al.*, 1965 ▸; Westman, 1965 ▸). However, it has long been known that the structure model of the γ-Al_8_Mn_5_ phase has some conflicts. For example, the Al_8_Mn_5_ phase was first reported with a hexa­gonal unit cell (*a* = 12.713 Å, *c* = 15.839 Å) and was believed to be associated with Al_8_Cr_5_ (Schubert *et al.*, 1960 ▸). Subsequently, this structure was checked with the unit-cell parameters *a* = 12.630 Å and *c* = 7.933 Å by powder diffraction patterns (Schonover & Mohanty, 1969 ▸). In another study, the low-temperature phase Al_8_Mn_5_ was analysed and considered as a hexa­gonal structure with unit-cell parameters *a* = 7.20 Å and *c* = 22.95 Å. In the me­antime, a high-temperature Al_8_Mn_5_ phase was reported (Koch *et al.*, 1960 ▸). Further studies were reported on the transformation from the high-temperature Al–Mn phase to the low-temperature Al_8_Mn_5_ phase, and on the measured metal concentrations, ranging from Mn_48_Al_52_ to Mn_37_Al_63_, with unit-cell parameters in the range *a* = 12.598–12.671 Å, and *c* = 7.911–7.942 Å, by powder diffraction patterns (Ellner, 1990 ▸). They reached the conclusion that the axial ratio *c*/*a* decreases while the molar fraction of aluminium increases. Very recently, the D8_10_–Al_8_Mn_5_ phase has been found to nucleate on B2–Al(Mn, Fe) particles in AZ91 magnesium alloys (Zeng *et al.*, 2018 ▸). The D8_10_–Al_8_Mn_5_ structure model closely resembles that described in the present work; however, its composition includes not only Al and Mn, but also other elements such as Fe and Mg.

Although the Al_8_Mn_5_ inter­metallic phase has been reported many times over many years, the atomic coordinates have not so far been determined accurately by single-crystal X-ray diffraction. In the present work, the crystal structure of the low-temperature γ-Al_8_Mn_5_-type phase with the refined chemical composition Al_8.6_Mn_4.4_ was determined by single-crystal X-ray diffraction measurements for the first time. Inter­metallic Al_8.6_Mn_4.4_ is a aluminium-rich phase compared to Mn_37_Al_63_, and its axial ratio, *c*/*a* = 0.624 is then slightly reduced (Mn_37_Al_63_: *c*/*a* = 0.626), in agreement with the results reported by Ellner (1990 ▸).

**Figure 1:**
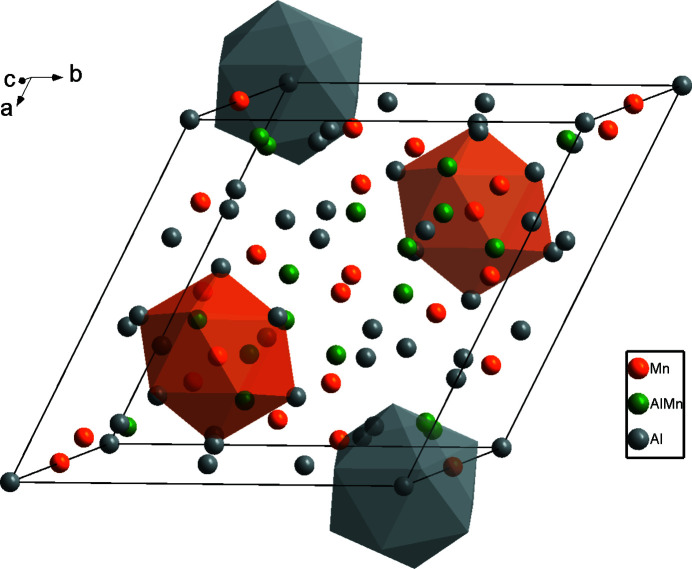
The crystal structure of Al_8.6_Mn_4.4_ with two Mn02 atoms and two Al04 atoms displayed with their coordination environments as polyhedra.

**Figure 2:**
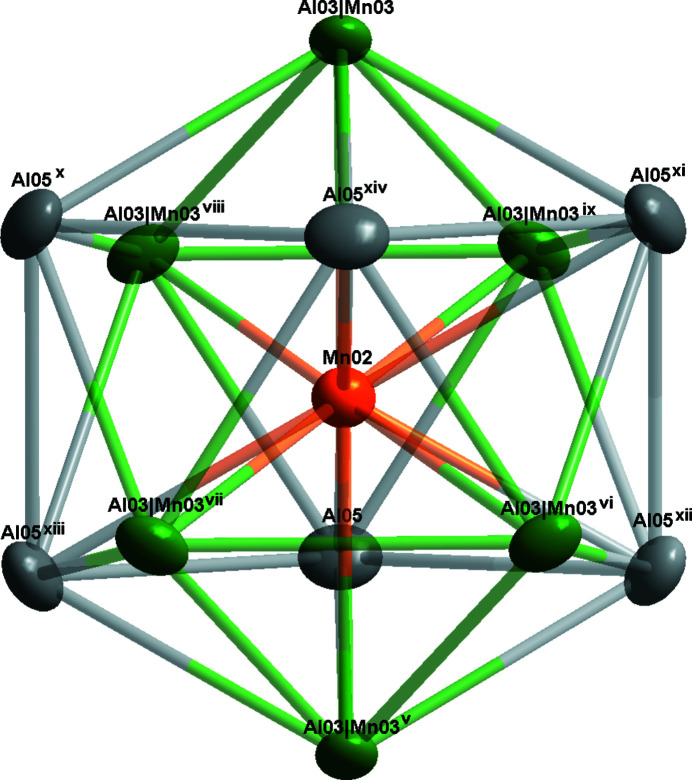
The environment of the Mn02 atom. Displacement ellipsoids are given at the 90% probability level. Symmetry codes: (v) −*x* + 4/3, −*y* + 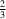 , −*z* + 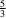 ; (vi) *y* + 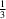 , −*x* + *y* + 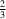 , −*z* + 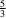 ; (vii) *x* − *y* + 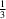 , *x* − 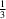 , −*z* + 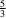 ; (viii) −*y* + 1*, x* − *y*, *z*; (ix) −*x* + *y* + 1, −*x* + 1, *z*; (x) −*x* + *y* + 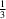 , −*x* + 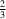 , *z* + 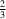 ; (xi) −*y* + 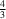 , *x* − *y* + 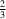 , *z* + 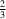 ; (xii) *x* − *y* + 1, *x*, −*z* + 1; (xiii) *y*, −*x* + *y*, −*z* + 1; (xiv) −*x* + 1, −*y* + 1, −*z* + 1.

**Figure 3:**
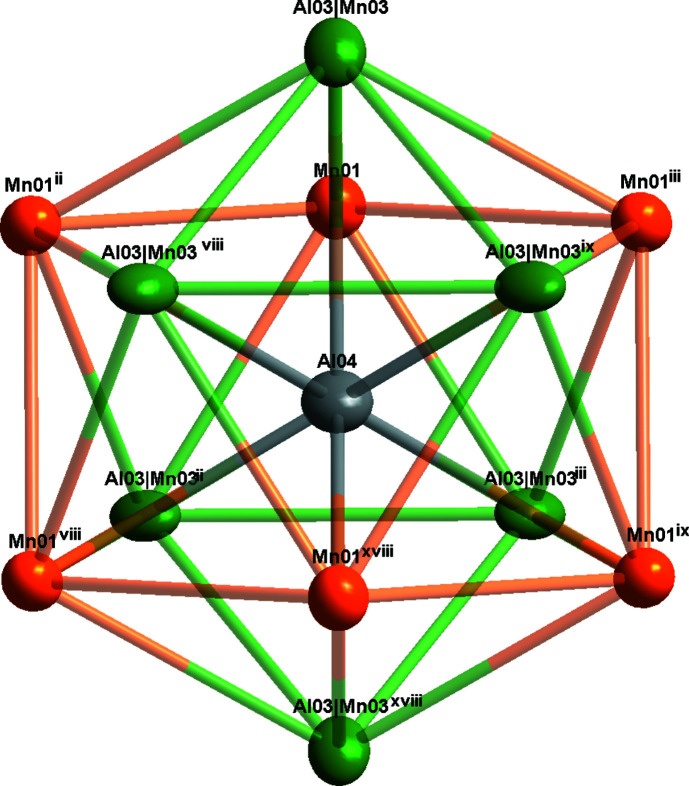
The environment of the Al04 atom. Displacement ellipsoids are given at the 90% probability level. Symmetry codes: (ii) *x* − *y* + 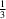 , *x* − 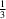 , −*z* + 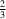 ; (iii) *y* + 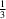 , −*x* + *y* + 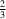 , −*z* + 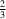 ; (viii) −*y* + 1, *x* − *y*, *z*; (ix) −*x* + *y* + 1, −*x* + 1, *z*; (xviii) −*x* + 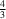 , −*y* + 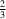 , −*z* + 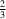 .

Fig. 1 ▸ shows the overall atomic distribution of Al_8.6_Mn_4.4_ in the unit cell. For simplicity, four distorted icosa­hedra are illustrated here, and the environment of the Mn02 atoms is shown in Fig. 2 ▸. The twelve vertices include six Al atoms (Al05) and six co-occupied Al/Mn sites (Al03/Mn03), where the refined site occupancies converged to 0.7 for Al03 and 0.3 for Mn03. In addition, the icosa­hedron centred at Al04 is shown in Fig. 3 ▸; it is constituted of six Mn atoms (Mn01) and six co-occupied Al/Mn sites (Al03/Mn03). In summary, these two icosa­hedra are packed together and form the main building blocks of Al_8.6_Mn_4.4_.

## Synthesis and crystallization

The high-purity elements Al (indicated purity 99.8%; 1.080 g) and Mn (indicated purity 99.96%; 1.100 g) were mixed in the stoichiometric ratio 2:1 and ground in an agate mortar. The blended powders were placed into a cemented carbide grinding mound of 9.6 mm diameter and pressed at 4 MPa for about 5 min. The obtained cylindrical block was crushed and a specimen weighing 50.55 mg was selected and subsequently loaded into the crucible of a Netzsch STA 449 C simultaneous thermal analysis instrument. The sample was heated up to 1250°C for 10 min with a heating rate of 20°C min^−1^, and then slowly cooled to 700°C with a cooling rate of 10°C min^−1^. Finally, the sample was cooled down to room temperature by switching off the furnace. Suitable pieces of single-crystal grains were selected from the educts for single-crystal X-ray diffraction experiments. Details of the EDS analysis are given in the supporting information.

## Refinement

Table 1 ▸ shows the details of data collection and structural refinement. Only one site is co-occupied by Al and Mn atoms (Al03/Mn03). Site occupancies were refined to 0.7 for Al03 and 0.3 for Mn03, and then fixed in the following least-squares cycles. Atoms sharing the same site were constrained to have the same coordinates and displacement parameters. The maximum and minimum residual electron densities in the last difference map are located 0.97 Å from atom Mn01 and 0.85 Å from atom Al05, respectively.

## Supplementary Material

Crystal structure: contains datablock(s) I. DOI: 10.1107/S2414314621009883/bh4065sup1.cif

Structure factors: contains datablock(s) I. DOI: 10.1107/S2414314621009883/bh4065Isup2.hkl

EDS analysis. DOI: 10.1107/S2414314621009883/bh4065sup3.docx

CCDC reference: 2111321

Additional supporting information:  crystallographic information; 3D view; checkCIF report

## Figures and Tables

**Table 1:** Experimental details

Crystal data	
Chemical formula	Al_8.6_Mn_4.4_
*M*_r_	473.76
Crystal system, space group	Trigonal, *R* 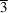 *m*:*H*
Temperature (K)	296
*a*, *c* (Å)	12.6751 (13), 7.9137 (9)
*V* (Å^3^)	1101.1 (3)
*Z*	6
Radiation type	Mo *K*α
μ (mm^−1^)	8.32
Crystal size (mm)	0.09 × 0.06 × 0.04

Data collection	
Diffractometer	Bruker D8 Venture Photon 100 CMOS
Absorption correction	Multi-scan (*SADABS*; Bruker, 2015 ▸)
*T*_min_, *T*_max_	0.494, 0.746
No. of measured, independent and observed [*I* > 2σ(*I*)] reflections	7162, 323, 298
*R*_int_	0.078
(sin θ/λ)_max_ (Å^−1^)	0.649

Refinement	
*R*[*F* ^2^ > 2σ(*F* ^2^)], *wR*(*F* ^2^), *S*	0.046, 0.125, 1.19
No. of reflections	323
No. of parameters	29
Δρ_max_, Δρ_min_ (e Å^−3^)	0.98, −1.23

Computer programs: *APEX3* and *SAINT* (Bruker, 2015 ▸), *SHELXT2014/5* (Sheldrick, 2015*a* ▸), *SHELXL2016/6* (Sheldrick, 2015*b* ▸), *DIAMOND* (Brandenburg & Putz, 2017 ▸) and *publCIF* (Westrip, 2010 ▸).

## full crystallographic data

### Crystal data

**Table:**

Al_8.6_Mn_4.4_	*D*_x_ = 4.287 Mg m^−^^3^
*M**_r_* = 473.76	Mo *K*α radiation, λ = 0.71073 Å
Trigonal, *R*3*m*:*H*	Cell parameters from 3882 reflections
*a* = 12.6751 (13) Å	θ = 3.2–30.4°
*c* = 7.9137 (9) Å	µ = 8.32 mm^−^^1^
*V* = 1101.1 (3) Å^3^	*T* = 296 K
*Z* = 6	Graininess, silver
*F*(000) = 1331	0.09 × 0.06 × 0.04 mm

### Data collection

**Table:**

Bruker D8 Venture Photon 100 CMOS diffractometer	298 reflections with *I* > 2σ(*I*)
φ and ω scans	*R*_int_ = 0.078
Absorption correction: multi-scan (SADABS; Bruker, 2015)	θ_max_ = 27.5°, θ_min_ = 3.2°
*T*_min_ = 0.494, *T*_max_ = 0.746	*h* = −16→16
7162 measured reflections	*k* = −15→16
323 independent reflections	*l* = −10→10

### Refinement

**Table:**

Refinement on *F*^2^	Primary atom site location: dual
Least-squares matrix: full	Secondary atom site location: difference Fourier map
*R*[*F*^2^ > 2σ(*F*^2^)] = 0.046	*w* = 1/[σ^2^(*F*_o_^2^) + (0.0472*P*)^2^ + 72.2358*P*] where *P* = (*F*_o_^2^ + 2*F*_c_^2^)/3
*wR*(*F*^2^) = 0.125	(Δ/σ)_max_ < 0.001
*S* = 1.19	Δρ_max_ = 0.98 e Å^−^^3^
323 reflections	Δρ_min_ = −1.23 e Å^−^^3^
29 parameters	Extinction correction: SHELXL-2016/6 (Sheldrick, 2015b), Fc^*^=kFc[1+0.001xFc^2^λ^3^/sin(2θ)]^-1/4^
0 restraints	Extinction coefficient: 0.0015 (3)

### Fractional atomic coordinates and isotropic or equivalent isotropic displacement parameters (Å^2^)

**Table:**

	*x*	*y*	*z*	*U*_iso_*/*U*_eq_	Occ. (<1)
Mn01	0.54960 (7)	0.45040 (7)	0.26408 (19)	0.0082 (5)	
Mn02	0.666667	0.333333	0.833333	0.0082 (8)	
Al03	0.59276 (11)	0.40724 (11)	0.5828 (3)	0.0082 (6)	0.7
Mn03	0.59276 (11)	0.40724 (11)	0.5828 (3)	0.0082 (6)	0.3
Al04	0.666667	0.333333	0.333333	0.0090 (15)	
Al05	0.45078 (13)	0.54922 (13)	0.0926 (4)	0.0101 (7)	
Al06	0.333333	0.2900 (3)	0.166667	0.0151 (8)	

### Atomic displacement parameters (Å^2^)

**Table:**

	*U*^11^	*U*^22^	*U*^33^	*U*^12^	*U*^13^	*U*^23^
Mn01	0.0079 (6)	0.0079 (6)	0.0085 (8)	0.0038 (6)	−0.0004 (3)	0.0004 (3)
Mn02	0.0086 (11)	0.0086 (11)	0.0072 (17)	0.0043 (6)	0.000	0.000
Al03	0.0090 (9)	0.0090 (9)	0.0046 (10)	0.0031 (9)	−0.0011 (4)	0.0011 (4)
Mn03	0.0090 (9)	0.0090 (9)	0.0046 (10)	0.0031 (9)	−0.0011 (4)	0.0011 (4)
Al04	0.011 (2)	0.011 (2)	0.006 (3)	0.0053 (11)	0.000	0.000
Al05	0.0127 (12)	0.0127 (12)	0.0096 (14)	0.0098 (13)	0.0005 (5)	−0.0005 (5)
Al06	0.0121 (15)	0.0156 (12)	0.0163 (16)	0.0060 (8)	−0.0051 (12)	−0.0026 (6)

### Geometric parameters (Å, º)

**Table:**

Mn01—Al05	2.559 (3)	Mn02—Al05^xii^	2.644 (3)
Mn01—Al06	2.5824 (10)	Mn02—Al05^xiii^	2.644 (3)
Mn01—Al06^i^	2.5825 (10)	Mn02—Al05^xiv^	2.644 (3)
Mn01—Al04	2.6278 (15)	Al03—Al04	2.556 (2)
Mn01—Al03^ii^	2.6650 (17)	Al03—Al06^iii^	2.650 (3)
Mn01—Al03^iii^	2.6650 (17)	Al03—Al06^iv^	2.650 (3)
Mn01—Al06^iii^	2.667 (2)	Al03—Al05^xi^	2.655 (3)
Mn01—Al06^iv^	2.667 (2)	Al03—Al05^x^	2.655 (3)
Mn01—Al03	2.695 (3)	Al03—Al05^xiv^	2.741 (4)
Mn01—Mn01^iii^	2.7941 (18)	Al03—Al03^ix^	2.810 (4)
Mn01—Mn01^ii^	2.7941 (18)	Al03—Al03^viii^	2.810 (4)
Mn02—Al03^v^	2.562 (2)	Al05—Al05^xv^	2.611 (6)
Mn02—Al03^vi^	2.562 (2)	Al05—Al05^i^	2.832 (4)
Mn02—Al03^vii^	2.562 (2)	Al05—Al05^xvi^	2.832 (4)
Mn02—Al03^viii^	2.562 (2)	Al05—Al06^i^	2.909 (3)
Mn02—Al03^ix^	2.562 (2)	Al05—Al06	2.909 (3)
Mn02—Al03	2.562 (2)	Al06—Al06^iv^	2.804 (2)
Mn02—Al05^x^	2.644 (3)	Al06—Al06^xvii^	2.804 (2)
Mn02—Al05^xi^	2.644 (3)		
			
Al05—Mn01—Al06	68.92 (8)	Mn02—Al03—Al03^viii^	56.73 (4)
Al05—Mn01—Al06^i^	68.92 (8)	Al06^iii^—Al03—Al03^viii^	163.86 (5)
Al06—Mn01—Al06^i^	135.16 (12)	Al06^iv^—Al03—Al03^viii^	111.76 (7)
Al05—Mn01—Al04	160.01 (9)	Al05^xi^—Al03—Al03^viii^	108.17 (7)
Al06—Mn01—Al04	107.13 (8)	Al05^x^—Al03—Al03^viii^	58.04 (6)
Al06^i^—Mn01—Al04	107.13 (8)	Mn01^ii^—Al03—Al03^viii^	58.18 (5)
Al05—Mn01—Al03^ii^	106.00 (8)	Mn01^iii^—Al03—Al03^viii^	107.93 (5)
Al06—Mn01—Al03^ii^	60.63 (8)	Mn01—Al03—Al03^viii^	107.73 (5)
Al06^i^—Mn01—Al03^ii^	118.97 (8)	Al05^xiv^—Al03—Al03^viii^	107.58 (7)
Al04—Mn01—Al03^ii^	57.74 (5)	Al03^ix^—Al03—Al03^viii^	60.0
Al05—Mn01—Al03^iii^	106.00 (8)	Al03^xviii^—Al04—Al03^ii^	66.71 (8)
Al06—Mn01—Al03^iii^	118.97 (8)	Al03^xviii^—Al04—Al03^ix^	113.29 (8)
Al06^i^—Mn01—Al03^iii^	60.63 (8)	Al03^ii^—Al04—Al03^ix^	180.0
Al04—Mn01—Al03^iii^	57.74 (5)	Al03^xviii^—Al04—Al03^iii^	66.71 (8)
Al03^ii^—Mn01—Al03^iii^	63.64 (11)	Al03^ii^—Al04—Al03^iii^	66.71 (8)
Al05—Mn01—Al06^iii^	91.44 (7)	Al03^ix^—Al04—Al03^iii^	113.29 (8)
Al06—Mn01—Al06^iii^	130.82 (5)	Al03^xviii^—Al04—Al03^viii^	113.29 (8)
Al06^i^—Mn01—Al06^iii^	64.55 (7)	Al03^ii^—Al04—Al03^viii^	113.29 (8)
Al04—Mn01—Al06^iii^	104.67 (5)	Al03^ix^—Al04—Al03^viii^	66.71 (8)
Al03^ii^—Mn01—Al06^iii^	162.37 (7)	Al03^iii^—Al04—Al03^viii^	180.00 (10)
Al03^iii^—Mn01—Al06^iii^	109.58 (7)	Al03^xviii^—Al04—Al03	180.0
Al05—Mn01—Al06^iv^	91.43 (7)	Al03^ii^—Al04—Al03	113.29 (8)
Al06—Mn01—Al06^iv^	64.55 (7)	Al03^ix^—Al04—Al03	66.71 (8)
Al06^i^—Mn01—Al06^iv^	130.82 (5)	Al03^iii^—Al04—Al03	113.29 (8)
Al04—Mn01—Al06^iv^	104.67 (5)	Al03^viii^—Al04—Al03	66.71 (8)
Al03^ii^—Mn01—Al06^iv^	109.58 (7)	Al03^xviii^—Al04—Mn01^iii^	118.14 (3)
Al03^iii^—Mn01—Al06^iv^	162.37 (7)	Al03^ii^—Al04—Mn01^iii^	118.14 (3)
Al06^iii^—Mn01—Al06^iv^	71.75 (12)	Al03^ix^—Al04—Mn01^iii^	61.86 (3)
Al05—Mn01—Al03	142.61 (10)	Al03^iii^—Al04—Mn01^iii^	62.63 (6)
Al06—Mn01—Al03	111.28 (5)	Al03^viii^—Al04—Mn01^iii^	117.37 (6)
Al06^i^—Mn01—Al03	111.28 (5)	Al03—Al04—Mn01^iii^	61.86 (3)
Al04—Mn01—Al03	57.37 (6)	Al03^xviii^—Al04—Mn01^ii^	118.14 (3)
Al03^ii^—Mn01—Al03	105.61 (8)	Al03^ii^—Al04—Mn01^ii^	62.63 (6)
Al03^iii^—Mn01—Al03	105.61 (8)	Al03^ix^—Al04—Mn01^ii^	117.37 (6)
Al06^iii^—Mn01—Al03	59.22 (5)	Al03^iii^—Al04—Mn01^ii^	118.14 (3)
Al06^iv^—Mn01—Al03	59.23 (5)	Al03^viii^—Al04—Mn01^ii^	61.86 (3)
Al05—Mn01—Mn01^iii^	126.73 (2)	Al03—Al04—Mn01^ii^	61.86 (3)
Al06—Mn01—Mn01^iii^	164.30 (8)	Mn01^iii^—Al04—Mn01^ii^	115.77 (2)
Al06^i^—Mn01—Mn01^iii^	59.33 (7)	Al03^xviii^—Al04—Mn01^viii^	61.86 (3)
Al04—Mn01—Mn01^iii^	57.883 (11)	Al03^ii^—Al04—Mn01^viii^	61.86 (3)
Al03^ii^—Mn01—Mn01^iii^	109.05 (6)	Al03^ix^—Al04—Mn01^viii^	118.14 (3)
Al03^iii^—Mn01—Mn01^iii^	59.10 (5)	Al03^iii^—Al04—Mn01^viii^	117.37 (6)
Al06^iii^—Mn01—Mn01^iii^	56.38 (6)	Al03^viii^—Al04—Mn01^viii^	62.63 (6)
Al06^iv^—Mn01—Mn01^iii^	112.33 (9)	Al03—Al04—Mn01^viii^	118.14 (3)
Al03—Mn01—Mn01^iii^	58.06 (5)	Mn01^iii^—Al04—Mn01^viii^	180.0
Al05—Mn01—Mn01^ii^	126.73 (2)	Mn01^ii^—Al04—Mn01^viii^	64.23 (2)
Al06—Mn01—Mn01^ii^	59.33 (7)	Al03^xviii^—Al04—Mn01^ix^	61.86 (3)
Al06^i^—Mn01—Mn01^ii^	164.30 (8)	Al03^ii^—Al04—Mn01^ix^	117.37 (6)
Al04—Mn01—Mn01^ii^	57.883 (11)	Al03^ix^—Al04—Mn01^ix^	62.63 (6)
Al03^ii^—Mn01—Mn01^ii^	59.10 (5)	Al03^iii^—Al04—Mn01^ix^	61.86 (3)
Al03^iii^—Mn01—Mn01^ii^	109.05 (6)	Al03^viii^—Al04—Mn01^ix^	118.14 (3)
Al06^iii^—Mn01—Mn01^ii^	112.33 (9)	Al03—Al04—Mn01^ix^	118.14 (3)
Al06^iv^—Mn01—Mn01^ii^	56.38 (6)	Mn01^iii^—Al04—Mn01^ix^	64.23 (2)
Al03—Mn01—Mn01^ii^	58.06 (5)	Mn01^ii^—Al04—Mn01^ix^	180.0
Mn01^iii^—Mn01—Mn01^ii^	105.61 (6)	Mn01^viii^—Al04—Mn01^ix^	115.77 (2)
Al03^v^—Mn02—Al03^vi^	66.53 (8)	Al03^xviii^—Al04—Mn01	117.37 (6)
Al03^v^—Mn02—Al03^vii^	66.53 (8)	Al03^ii^—Al04—Mn01	61.86 (3)
Al03^vi^—Mn02—Al03^vii^	66.53 (8)	Al03^ix^—Al04—Mn01	118.14 (3)
Al03^v^—Mn02—Al03^viii^	113.47 (8)	Al03^iii^—Al04—Mn01	61.86 (3)
Al03^vi^—Mn02—Al03^viii^	180.0	Al03^viii^—Al04—Mn01	118.14 (3)
Al03^vii^—Mn02—Al03^viii^	113.47 (9)	Al03—Al04—Mn01	62.63 (6)
Al03^v^—Mn02—Al03^ix^	113.47 (8)	Mn01^iii^—Al04—Mn01	64.23 (2)
Al03^vi^—Mn02—Al03^ix^	113.47 (9)	Mn01^ii^—Al04—Mn01	64.23 (2)
Al03^vii^—Mn02—Al03^ix^	180.0	Mn01^viii^—Al04—Mn01	115.77 (2)
Al03^viii^—Mn02—Al03^ix^	66.53 (8)	Mn01^ix^—Al04—Mn01	115.77 (2)
Al03^v^—Mn02—Al03	180.0	Mn01—Al05—Al05^xv^	66.17 (13)
Al03^vi^—Mn02—Al03	113.47 (8)	Mn01—Al05—Mn02^xix^	135.17 (13)
Al03^vii^—Mn02—Al03	113.47 (8)	Al05^xv^—Al05—Mn02^xix^	158.7 (2)
Al03^viii^—Mn02—Al03	66.53 (8)	Mn01—Al05—Al03^xx^	147.49 (7)
Al03^ix^—Mn02—Al03	66.53 (8)	Al05^xv^—Al05—Al03^xx^	104.81 (15)
Al03^v^—Mn02—Al05^x^	118.71 (5)	Mn02^xix^—Al05—Al03^xx^	57.82 (7)
Al03^vi^—Mn02—Al05^x^	118.71 (5)	Mn01—Al05—Al03^xxi^	147.49 (7)
Al03^vii^—Mn02—Al05^x^	63.51 (9)	Al05^xv^—Al05—Al03^xxi^	104.81 (15)
Al03^viii^—Mn02—Al05^x^	61.29 (5)	Mn02^xix^—Al05—Al03^xxi^	57.82 (7)
Al03^ix^—Mn02—Al05^x^	116.49 (9)	Al03^xx^—Al05—Al03^xxi^	63.92 (12)
Al03—Mn02—Al05^x^	61.29 (5)	Mn01—Al05—Al03^xiv^	78.39 (10)
Al03^v^—Mn02—Al05^xi^	118.71 (5)	Al05^xv^—Al05—Al03^xiv^	144.6 (2)
Al03^vi^—Mn02—Al05^xi^	63.51 (9)	Mn02^xix^—Al05—Al03^xiv^	56.78 (8)
Al03^vii^—Mn02—Al05^xi^	118.71 (5)	Al03^xx^—Al05—Al03^xiv^	105.11 (11)
Al03^viii^—Mn02—Al05^xi^	116.49 (9)	Al03^xxi^—Al05—Al03^xiv^	105.11 (11)
Al03^ix^—Mn02—Al05^xi^	61.29 (5)	Mn01—Al05—Mn01^xv^	122.19 (11)
Al03—Mn02—Al05^xi^	61.29 (5)	Al05^xv^—Al05—Mn01^xv^	56.02 (12)
Al05^x^—Mn02—Al05^xi^	115.24 (5)	Mn02^xix^—Al05—Mn01^xv^	102.64 (10)
Al03^v^—Mn02—Al05^xii^	61.29 (5)	Al03^xx^—Al05—Mn01^xv^	58.13 (7)
Al03^vi^—Mn02—Al05^xii^	61.29 (5)	Al03^xxi^—Al05—Mn01^xv^	58.13 (7)
Al03^vii^—Mn02—Al05^xii^	116.49 (9)	Al03^xiv^—Al05—Mn01^xv^	159.42 (13)
Al03^viii^—Mn02—Al05^xii^	118.71 (5)	Mn01—Al05—Al05^i^	99.58 (11)
Al03^ix^—Mn02—Al05^xii^	63.51 (9)	Al05^xv^—Al05—Al05^i^	127.52 (4)
Al03—Mn02—Al05^xii^	118.71 (5)	Mn02^xix^—Al05—Al05^i^	57.62 (2)
Al05^x^—Mn02—Al05^xii^	180.0	Al03^xx^—Al05—Al05^i^	109.39 (8)
Al05^xi^—Mn02—Al05^xii^	64.76 (5)	Al03^xxi^—Al05—Al05^i^	59.82 (8)
Al03^v^—Mn02—Al05^xiii^	61.29 (5)	Al03^xiv^—Al05—Al05^i^	56.87 (10)
Al03^vi^—Mn02—Al05^xiii^	116.49 (9)	Mn01^xv^—Al05—Al05^i^	114.36 (13)
Al03^vii^—Mn02—Al05^xiii^	61.29 (5)	Mn01—Al05—Al05^xvi^	99.58 (11)
Al03^viii^—Mn02—Al05^xiii^	63.50 (9)	Al05^xv^—Al05—Al05^xvi^	127.52 (4)
Al03^ix^—Mn02—Al05^xiii^	118.71 (5)	Mn02^xix^—Al05—Al05^xvi^	57.62 (2)
Al03—Mn02—Al05^xiii^	118.71 (5)	Al03^xx^—Al05—Al05^xvi^	59.82 (8)
Al05^x^—Mn02—Al05^xiii^	64.76 (5)	Al03^xxi^—Al05—Al05^xvi^	109.39 (8)
Al05^xi^—Mn02—Al05^xiii^	180.0	Al03^xiv^—Al05—Al05^xvi^	56.87 (10)
Al05^xii^—Mn02—Al05^xiii^	115.23 (5)	Mn01^xv^—Al05—Al05^xvi^	114.36 (13)
Al03^v^—Mn02—Al05^xiv^	116.49 (9)	Al05^i^—Al05—Al05^xvi^	104.07 (14)
Al03^vi^—Mn02—Al05^xiv^	61.29 (5)	Mn01—Al05—Al06^i^	55.92 (5)
Al03^vii^—Mn02—Al05^xiv^	61.29 (5)	Al05^xv^—Al05—Al06^i^	70.76 (8)
Al03^viii^—Mn02—Al05^xiv^	118.71 (5)	Mn02^xix^—Al05—Al06^i^	118.49 (7)
Al03^ix^—Mn02—Al05^xiv^	118.71 (5)	Al03^xx^—Al05—Al06^i^	153.69 (11)
Al03—Mn02—Al05^xiv^	63.51 (9)	Al03^xxi^—Al05—Al06^i^	91.59 (6)
Al05^x^—Mn02—Al05^xiv^	64.77 (5)	Al03^xiv^—Al05—Al06^i^	89.87 (8)
Al05^xi^—Mn02—Al05^xiv^	64.77 (5)	Mn01^xv^—Al05—Al06^i^	101.71 (7)
Al05^xii^—Mn02—Al05^xiv^	115.23 (5)	Al05^i^—Al05—Al06^i^	60.87 (6)
Al05^xiii^—Mn02—Al05^xiv^	115.23 (5)	Al05^xvi^—Al05—Al06^i^	143.81 (17)
Al04—Al03—Mn02	101.29 (8)	Mn01—Al05—Al06	55.92 (5)
Al04—Al03—Al06^iii^	107.27 (7)	Al05^xv^—Al05—Al06	70.75 (8)
Mn02—Al03—Al06^iii^	132.71 (7)	Mn02^xix^—Al05—Al06	118.49 (7)
Al04—Al03—Al06^iv^	107.27 (7)	Al03^xx^—Al05—Al06	91.59 (6)
Mn02—Al03—Al06^iv^	132.71 (7)	Al03^xxi^—Al05—Al06	153.69 (11)
Al06^iii^—Al03—Al06^iv^	72.31 (13)	Al03^xiv^—Al05—Al06	89.87 (8)
Al04—Al03—Al05^xi^	109.07 (7)	Mn01^xv^—Al05—Al06	101.71 (7)
Mn02—Al03—Al05^xi^	60.89 (7)	Al05^i^—Al05—Al06	143.81 (17)
Al06^iii^—Al03—Al05^xi^	74.36 (8)	Al05^xvi^—Al05—Al06	60.87 (6)
Al06^iv^—Al03—Al05^xi^	136.29 (11)	Al06^i^—Al05—Al06	110.28 (11)
Al04—Al03—Al05^x^	109.07 (7)	Mn01^xvi^—Al06—Mn01	150.27 (16)
Mn02—Al03—Al05^x^	60.89 (7)	Mn01^xvi^—Al06—Al03^xvii^	61.23 (5)
Al06^iii^—Al03—Al05^x^	136.30 (11)	Mn01—Al06—Al03^xvii^	141.63 (9)
Al06^iv^—Al03—Al05^x^	74.36 (8)	Mn01^xvi^—Al06—Al03^ii^	141.63 (9)
Al05^xi^—Al03—Al05^x^	114.53 (15)	Mn01—Al06—Al03^ii^	61.23 (5)
Al04—Al03—Mn01^ii^	60.40 (5)	Al03^xvii^—Al06—Al03^ii^	107.69 (13)
Mn02—Al03—Mn01^ii^	109.51 (6)	Mn01^xvi^—Al06—Mn01^xvii^	64.29 (6)
Al06^iii^—Al03—Mn01^ii^	117.24 (9)	Mn01—Al06—Mn01^xvii^	137.27 (7)
Al06^iv^—Al03—Mn01^ii^	58.15 (5)	Al03^xvii^—Al06—Mn01^xvii^	60.90 (8)
Al05^xi^—Al03—Mn01^ii^	165.50 (11)	Al03^ii^—Al06—Mn01^xvii^	78.16 (9)
Al05^x^—Al03—Mn01^ii^	64.09 (7)	Mn01^xvi^—Al06—Mn01^ii^	137.27 (7)
Al04—Al03—Mn01^iii^	60.40 (5)	Mn01—Al06—Mn01^ii^	64.29 (6)
Mn02—Al03—Mn01^iii^	109.51 (6)	Al03^xvii^—Al06—Mn01^ii^	78.16 (9)
Al06^iii^—Al03—Mn01^iii^	58.14 (5)	Al03^ii^—Al06—Mn01^ii^	60.90 (8)
Al06^iv^—Al03—Mn01^iii^	117.23 (9)	Mn01^xvii^—Al06—Mn01^ii^	108.25 (12)
Al05^xi^—Al03—Mn01^iii^	64.09 (7)	Mn01^xvi^—Al06—Al06^iv^	111.18 (4)
Al05^x^—Al03—Mn01^iii^	165.50 (11)	Mn01—Al06—Al06^iv^	59.19 (9)
Mn01^ii^—Al03—Mn01^iii^	113.26 (10)	Al03^xvii^—Al06—Al06^iv^	94.07 (10)
Al04—Al03—Mn01	60.00 (6)	Al03^ii^—Al06—Al06^iv^	106.01 (5)
Mn02—Al03—Mn01	161.29 (11)	Mn01^xvii^—Al06—Al06^iv^	154.14 (16)
Al06^iii^—Al03—Mn01	59.88 (6)	Mn01^ii^—Al06—Al06^iv^	56.26 (3)
Al06^iv^—Al03—Mn01	59.88 (6)	Mn01^xvi^—Al06—Al06^xvii^	59.19 (9)
Al05^xi^—Al03—Mn01	122.45 (7)	Mn01—Al06—Al06^xvii^	111.18 (4)
Al05^x^—Al03—Mn01	122.45 (7)	Al03^xvii^—Al06—Al06^xvii^	106.00 (5)
Mn01^ii^—Al03—Mn01	62.84 (5)	Al03^ii^—Al06—Al06^xvii^	94.07 (10)
Mn01^iii^—Al03—Mn01	62.84 (5)	Mn01^xvii^—Al06—Al06^xvii^	56.25 (3)
Al04—Al03—Al05^xiv^	161.01 (12)	Mn01^ii^—Al06—Al06^xvii^	154.14 (16)
Mn02—Al03—Al05^xiv^	59.72 (8)	Al06^iv^—Al06—Al06^xvii^	145.82 (19)
Al06^iii^—Al03—Al05^xiv^	87.92 (8)	Mn01^xvi^—Al06—Al05	97.07 (11)
Al06^iv^—Al03—Al05^xiv^	87.92 (8)	Mn01—Al06—Al05	55.16 (8)
Al05^xi^—Al03—Al05^xiv^	63.31 (7)	Al03^xvii^—Al06—Al05	154.93 (11)
Al05^x^—Al03—Al05^xiv^	63.31 (7)	Al03^ii^—Al06—Al05	97.17 (6)
Mn01^ii^—Al03—Al05^xiv^	123.08 (5)	Mn01^xvii^—Al06—Al05	123.49 (8)
Mn01^iii^—Al03—Al05^xiv^	123.08 (5)	Mn01^ii^—Al06—Al05	118.16 (7)
Mn01—Al03—Al05^xiv^	139.00 (12)	Al06^iv^—Al06—Al05	81.81 (14)
Al04—Al03—Al03^ix^	56.65 (4)	Al06^xvii^—Al06—Al05	68.22 (8)
Mn02—Al03—Al03^ix^	56.73 (4)	Mn01^xvi^—Al06—Al05^xvi^	55.15 (8)
Al06^iii^—Al03—Al03^ix^	111.75 (7)	Mn01—Al06—Al05^xvi^	97.07 (11)
Al06^iv^—Al03—Al03^ix^	163.86 (5)	Al03^xvii^—Al06—Al05^xvi^	97.18 (6)
Al05^xi^—Al03—Al03^ix^	58.04 (6)	Al03^ii^—Al06—Al05^xvi^	154.93 (11)
Al05^x^—Al03—Al03^ix^	108.17 (7)	Mn01^xvii^—Al06—Al05^xvi^	118.16 (7)
Mn01^ii^—Al03—Al03^ix^	107.93 (5)	Mn01^ii^—Al06—Al05^xvi^	123.49 (8)
Mn01^iii^—Al03—Al03^ix^	58.18 (5)	Al06^iv^—Al06—Al05^xvi^	68.22 (8)
Mn01—Al03—Al03^ix^	107.73 (5)	Al06^xvii^—Al06—Al05^xvi^	81.82 (14)
Al05^xiv^—Al03—Al03^ix^	107.58 (7)	Al05—Al06—Al05^xvi^	58.26 (12)
Al04—Al03—Al03^viii^	56.65 (4)		

Symmetry codes: (i) *x*−*y*+2/3, *x*+1/3, −*z*+1/3; (ii) *x*−*y*+1/3, *x*−1/3, −*z*+2/3; (iii) *y*+1/3, −*x*+*y*+2/3, −*z*+2/3; (iv) −*y*+2/3, *x*−*y*+1/3, *z*+1/3; (v) −*x*+4/3, −*y*+2/3, −*z*+5/3; (vi) *y*+1/3, −*x*+*y*+2/3, −*z*+5/3; (vii) *x*−*y*+1/3, *x*−1/3, −*z*+5/3; (viii) −*y*+1, *x*−*y*, *z*; (ix) −*x*+*y*+1, −*x*+1, *z*; (x) −*x*+*y*+1/3, −*x*+2/3, *z*+2/3; (xi) −*y*+4/3, *x*−*y*+2/3, *z*+2/3; (xii) *x*−*y*+1, *x*, −*z*+1; (xiii) *y*, −*x*+*y*, −*z*+1; (xiv) −*x*+1, −*y*+1, −*z*+1; (xv) −*x*+1, −*y*+1, −*z*; (xvi) *y*−1/3, −*x*+*y*+1/3, −*z*+1/3; (xvii) −*x*+*y*+1/3, −*x*+2/3, *z*−1/3; (xviii) −*x*+4/3, −*y*+2/3, −*z*+2/3; (xix) *x*−1/3, *y*+1/3, *z*−2/3; (xx) −*y*+2/3, *x*−*y*+1/3, *z*−2/3; (xxi) −*x*+*y*+2/3, −*x*+4/3, *z*−2/3.
