# Supplementary material for: The structure of the aluminium-abundant γ-brass-type Al8.6Mn4.4
Source: IUCrdata. 2021 Sep 24;6(Pt 9):x210988. doi: 10.1107/S2414314621009883 (PMC9462374; doi:10.1107/S2414314621009883)
Supplement: Supplementary file 3 [file x-06-x210988-sup3.docx]

**SUPPLEMENTARY MATERIALS:**

**The structrue of aluminium-abundant *γ*-brass-type Al_1.95_Mn**

**Qifa Hu, Bin Wen and Changzeng Fan***

State Key Laboratory of Metastable Materials Science and Technology, Yanshan University,

Qinhuangdao 066004, P.R. China

*Correspondence email: [chzfan@ysu.edu.cn](mailto:chzfan@ysu.edu.cn)

In order to guide the refinement process, the chemical compositions were examined quantitatively by energy dispersive X-ray spectroscopy (EDS) analysis attached to a Hitachi S-3400N SEM. The examined points are designated in Fig. S1 and the corresponding EDS results are listed in Table S1. The fluctuation of chemical compositions is probably caused by the tilt of single crystal specimen; The detected carbon is probably attributed to the conductive adhesives and glues. The detected oxygen is probably attributed to oxidation afterwards. The ratio of Al and Mn are also calculated and shown in the Tab. S1.


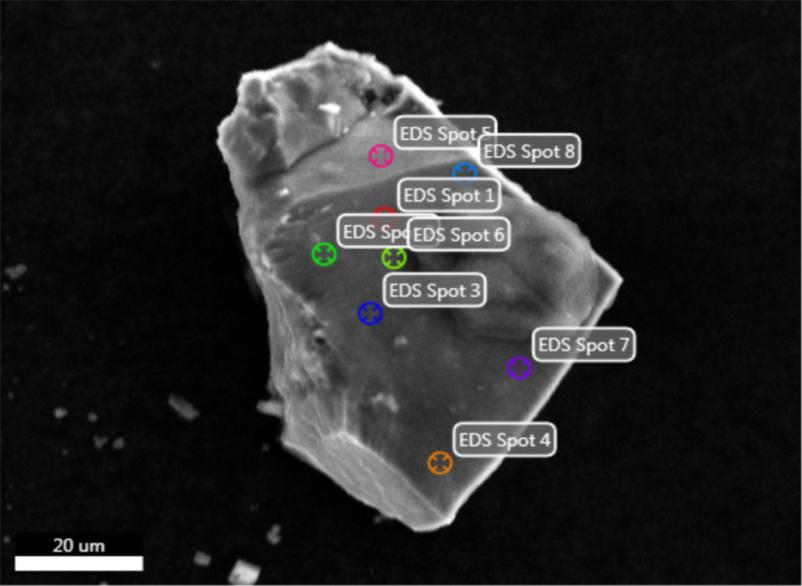


**Fig. S1** The measured single-crystal specimen

**Tab. S1** EDS results for selected points

|  | Element | Weight(%) | Atomic(%) | Error(%) | Al : Mn |
| --- | --- | --- | --- | --- | --- |
| Spot 1 | Al K | 47.63 | 64.93 | 6.07 | 1.85 : 1 |
|  | Mn K | 52.37 | 35.07 | 2.29 |  |
| Spot 2 | C K | 43.45 | 67.58 | 10.02 | 1.75 : 1 |
|  | O K | 6.01 | 7.02 | 12.13 |  |
|  | Al K | 23.33 | 16.15 | 5.07 |  |
|  | Mn K | 27.20 | 9.25 | 2.47 |  |
| Spot 3 | C K | 45.42 | 69.96 | 9.83 | 1.59 : 1 |
|  | O K | 4.97 | 5.75 | 12.54 |  |
|  | Al K | 21.77 | 14.92 | 5.15 |  |
|  | Mn K | 27.84 | 9.38 | 2.42 |  |
| Spot 4 | C K | 57.04 | 78.41 | 9.05 | 14.1 : 1 |
|  | O K | 5.68 | 5.86 | 12.92 |  |
|  | Al K | 14.86 | 9.09 | 4.96 |  |
|  | Mn K | 21.45 | 6.45 | 2.75 |  |
| Spot 5 | C K | 13.68 | 32.11 | 13.85 | **2.15 : 1** |
|  | Al K | 44.38 | 46.37 | 5.53 |  |
|  | Mn K | 41.93 | 21.52 | 2.25 |  |
| Spot 6 | C K | 11.98 | 29.66 | 14.19 | 1.74 : 1 |
|  | Al K | 40.51 | 44.63 | 5.90 |  |
|  | Mn K | 47.51 | 25.71 | 2.24 |  |
| Spot 7 | C K | 41.82 | 69.85 | 10.10 | 1.53 : 1 |
|  | O K | 5.43 | 6.52 | 12.30 |  |
|  | Al K | 22.64 | 16.11 | 5.31 |  |
|  | Mn K | 30.10 | 10.52 | 2.46 |  |
| Spot 8 | C K | 23.57 | 48.88 | 11.86 | 1.73 : 1 |
|  | Al K | 35.06 | 32.37 | 5.63 |  |
|  | Mn K | 41.36 | 18.75 | 2.23 |  |
